# A novel subnetwork alignment approach predicts new components of the cell cycle regulatory apparatus in *Plasmodium falciparum*

**DOI:** 10.1186/1471-2105-14-S12-S2

**Published:** 2013-09-24

**Authors:** Hong Cai, Changjin Hong, Timothy G Lilburn, Armando L Rodriguez, Sheng Chen, Jianying Gu, Rui Kuang, Yufeng Wang

**Affiliations:** 1 Department of Biology, University of Texas at San Antonio, San Antonio, TX 78249, USA; 2 Department of Computer Science and Engineering, University of Minnesota Twin Cities, Minneapolis, MN 55455, USA; 3 Department of Bacteriology, American Type Culture Collection, Manassas, VA 20110, USA; 4 Department of Computer Science, University of Texas at San Antonio, San Antonio, TX 78249, USA; 5 Department of Biology, College of Staten Island, City University of New York, Staten Island, NY 10314, USA; 6 South Texas Center for Emerging Infectious Diseases, University of Texas at San Antonio, San Antonio, TX 78249, USA

## Abstract

**Background:**

According to the World Health organization, half the world's population is at risk of contracting malaria. They estimated that in 2010 there were 219 million cases of malaria, resulting in 660,000 deaths and an enormous economic burden on the countries where malaria is endemic. The adoption of various high-throughput genomics-based techniques by malaria researchers has meant that new avenues to the study of this disease are being explored and new targets for controlling the disease are being developed. Here, we apply a novel neighborhood subnetwork alignment approach to identify the interacting elements that help regulate the cell cycle of the malaria parasite *Plasmodium falciparum*.

**Results:**

Our novel subnetwork alignment approach was used to compare networks in *Escherichia coli *and *P. falciparum*. Some 574 *P. falciparum *proteins were revealed as functional orthologs of known cell cycle proteins in *E. coli*. Over one third of these predicted functional orthologs were annotated as "conserved *Plasmodium *proteins" or "putative uncharacterized proteins" of unknown function. The predicted functionalities included cyclins, kinases, surface antigens, transcriptional regulators and various functions related to DNA replication, repair and cell division.

**Conclusions:**

The results of our analysis demonstrate the power of our subnetwork alignment approach to assign functionality to previously unannotated proteins. Here, the focus was on proteins involved in cell cycle regulation. These proteins are involved in the control of diverse aspects of the parasite lifecycle and of important aspects of pathogenesis.

## Background

Written descriptions of the symptoms of malaria have existed for over 4,000 years and evidence for the existence of the genus *Plasmodium *has been recovered from amber approximately 30 million years old [1]. Thus, the disease has probably evolved alongside its hosts since the emergence of the first humans in Africa. In 2010, it was estimate that 660,000 people died from malaria. This estimate probably represents a conservative number, as reporting of the disease is extremely variable from one region to another; generally, the regions with the highest incidence of malaria also have the weakest mechanisms for reporting and recording cases.

Malaria is caused by protozoan parasites from the Genus *Plasmodium*. Different species tend to infect different host species. Five species infect humans; the two most widespread species are *P. vivax *and *P. falciparum*. The latter species is the most lethal. *P. falciparum *has a complex life cycle that spans the arthropod vector and human host. Upon transfer from the vector to the human host, the parasite first infects the liver. After maturation in the liver, the parasite infects red blood cells. In this so-called RBC stage, the symptoms of malaria become acute.

A number of antimicrobial drugs have been developed over the years, notably chloroquine and artemisinin. However, in the past decades, the effectiveness of all these drugs has been significantly reduced due to the evolution of drug-resistant parasites, with the exception of artemisinin. Recently, however, evidence has emerged that resistance to artemisinin has appeared and is beginning to spread. Therefore, it is essential that new drug targets be identified and the development of new genomics-based technologies is key to this task. Genome sequences from *P. falciparum *[2] and other *Plasmodium *spp. [3-7] have been completed and these have facilitated numerous studies on, for example, parasite transcription [8-19], translation [20-29], metabolism [30-34], protein-protein interactions [35-38], and epigenetic regulation [39-42]. The data from these studies have, in turn, laid the groundwork for systems biology oriented studies of the networks associated with parasite development, survival, pathogenesis, and virulence [43-46].

Network alignment is a popular systems biology method [47-55]. However, because the malaria parasite is only distantly related to other, more completely understood model organisms, the utility of this approach may be cast in doubt. About 60% of the open reading frames in *P. falciparum *are annotated as "hypothetical proteins" [2] simply because homology transfer of information about individual proteins is not possible across extended evolutionary distances. To tackle this problem, we recently developed a neighborhood subnetwork alignment algorithm [56], which is focused on the similarities between functional modules, in other words, on the interactions among proteins rather than on individual proteins. We define a neighborhood subnetwork as the set of nodes (proteins) reachable from a central protein via a small number of edges in a protein-protein interaction (PPI) network. A proof-of-concept study predicted previously unrecognized transcriptional regulators involved in diverse facets of the parasite life cycle [43]. In this paper, we use the subnetwork alignment approach to uncover candidate proteins with roles in cell cycle regulation, several of which are potential drug targets. As our knowledge of the mechanics of the cell cycle deepens, so will our ability to influence parasite survival in the host and our ability to identify key drug targets.

## Results and discussion

### Neighborhood subnetwork alignments predicted 574 proteins that are associated with cell cycle regulation in malaria parasite

The cell cycle of the malaria parasite differs significantly from that of other model eukaryotic organisms. There is no direct correspondence between schizogony, during which the parasite undergoes multiplication, and the typical G1, S, G2 and M phases of the cell cycle in crown eukaryotes. In addition, the parasite's cell cycle features asynchronous nuclear divisions, organellar segregation, and morphogenesis of daughter merozoites. A thorough sequence similarity-based search by Doerig and Chakrabarti predicted a list of proteins that might be involved in the cell cycle [57], including cyclins, cyclin-dependent kinases, proteins critical for cell division and signal transduction. In a previous study, we used a variational Bayesian expectation maximization (VBEM) approach to reveal the dynamics of the parasite cell cycle network, and to infer regulatory relationships based on time-series transcriptomic data [58]. The results from that study exposed gaps in our cell cycle network model. Here we use our subnetwork alignment approach to try and fill these gaps.

We predicted that 574 proteins in *P. falciparum *were functional orthologs of known cell cycle proteins in *E. coli *(Additional File 1). Over 34% of these predicted functional orthologs were annotated as "conserved *Plasmodium *proteins" or "putative uncharacterized proteins" of unknown function.

### The set of functional orthologs is involved in key biological processes

Table 1 shows representative functional categories predicted for the cell cycle-associated protein set as revealed by Gene Ontology (GO) enrichment analysis. These functional categories are part of some of the most important mechanisms governing the growth and survival of the parasite. Some of the more interesting functional groups are discussed in the following sections.

**Table 1:** Representative *P. falciparum *proteins that were predicted to be involved in cell cycle regulatory network.

Functional category	PlasmoDB accession number	Annotation
Cyclin	PFL1330c	Cyclin-related protein, Pfcyc-2

Cell differentiation	PFE0375w	cell differentiation protein, putative (CAF40)

Chromosome organization	PFE0450w	Chromosome condensation protein, putative
	PF11_0062	Histone H2B

Mitosis	PF13_0050	HORMA domain protein, putative

DNA repair	MAL7P1.145	Mismatch repair protein pms1 homologue, putative;
	PF10_0114	DNA repair protein RAD23, putative
	PF08_0126	DNA repair protein rad54, putative

DNA replication	PF07_0023	DNA replication licensing factor mcm7 homologue, putative
	PFL0580w	DNA replication licensing factor MCM5, putative
	MAL7P1.21	Origin recognition complex subunit 2, putative
	PFE1345c	Minichromosome maintenance protein 3, putative

Regulation of cell cycle	PF07_0047	AAA family ATPase, CDC48 subfamily (Cdc48)
	PFL1925w	Cell division protein FtsH, putative

Protein phosphorylation	PFC0105w	Serine/threonine protein kinase, putative
	MAL13P1.278	Serine/threonine protein kinase, putative
	PF14_0294	Mitogen-activated protein kinase 1
	PFC0755c	Protein kinase, putative
	PF11_0464	Ser/Thr protein kinase, putative
	PF11_0156	Ser/Thr protein kinase
	PF11_0239	Calcium-dependent protein kinase, putative
	PFL1370w	NIMA-related protein kinase, Pfnek-1

Proteolysis	PF14_0517	Peptidase, putative
	MAL13P1.184	Endopeptidase, putative
	PFL1635w	Ulp1 protease, putative
	PF10_0150	Methionine aminopeptidase

Cytoskeleton	MAL8P1.146	filament assembling protein, putative

Heat shock	PFI0875w	Heat shock protein 70 (HSP70) homologue
	PFL0565w	Heat shock protein DNAJ homologue Pfj4
	PF11_0351	Heat shock protein hsp70 homologue
	PF11_0188	Heat shock protein 90, putative
	PF07_0029	Heat shock protein 86
	PF08_0054	Heat shock 70 kDa protein
	PFB0595w	Heat shock 40 kDa protein, putative
	PFI0355c	ATP-dependent heat shock protein, putative

Pathogenesis	PFC0005w	Erythrocyte membrane protein 1, PfEMP1
	PFI0005w	Erythrocyte membrane protein 1, PfEMP1
	PFD0005w	Erythrocyte membrane protein 1, PfEMP1
	PF08_0103	Erythrocyte membrane protein 1, PfEMP1
	PFL0935c	Erythrocyte membrane protein 1, PfEMP1
	PFL0005w	Erythrocyte membrane protein 1, PfEMP1
	PFB1055c	Erythrocyte membrane protein 1, PfEMP1
	PFI1830c	Erythrocyte membrane protein 1, PfEMP1

Microtubule cytoskeleton organization and activity	PFC0165w	Spindle pole body protein, putative
	PF07_0104	Kinesin-like protein, putative

Transcriptional regulation	PF10_0143	Transcriptional coactivator ADA2 (ADA2)
	PFD0985w	AP2/ERF domain-containing protein PFD0985w
	PFL1085w	Transcription factor with AP2 domain, putative
	PF11_0442	Transcription factor with AP2 domain, putative
	PFE0840c	Transcription factor with AP2 domain, putative
	PF07_0126	Transcription factor with AP2 domain, putative
	PF10_0075	Transcription factor with AP2 domain, putative
	PFL1900w	Transcription factor with AP2 domain, putative
	PFL0465c	Zinc finger transcription factor (Krox1)

#### 1. Cyclin

Our subnetwork alignment approach predicted PFL1330c to be a putative cyclin [58]. Cyclins are a family of proteins with expression levels that oscillate during the cell cycle; the synthesis and degradation of cyclins control the activity of cyclin-dependent kinases and accurate transition of key cell cycle points. Yeast two-hybrid (Y2H) experiments [37] have shown that PFL1330c has physical interaction with an apical sushi protein (ASP) (PFD0295c), which has an adhesive "sushi" domain and thought to have a role in the merozoite invasion process.

#### 2. Kinases

**Figure 1:**
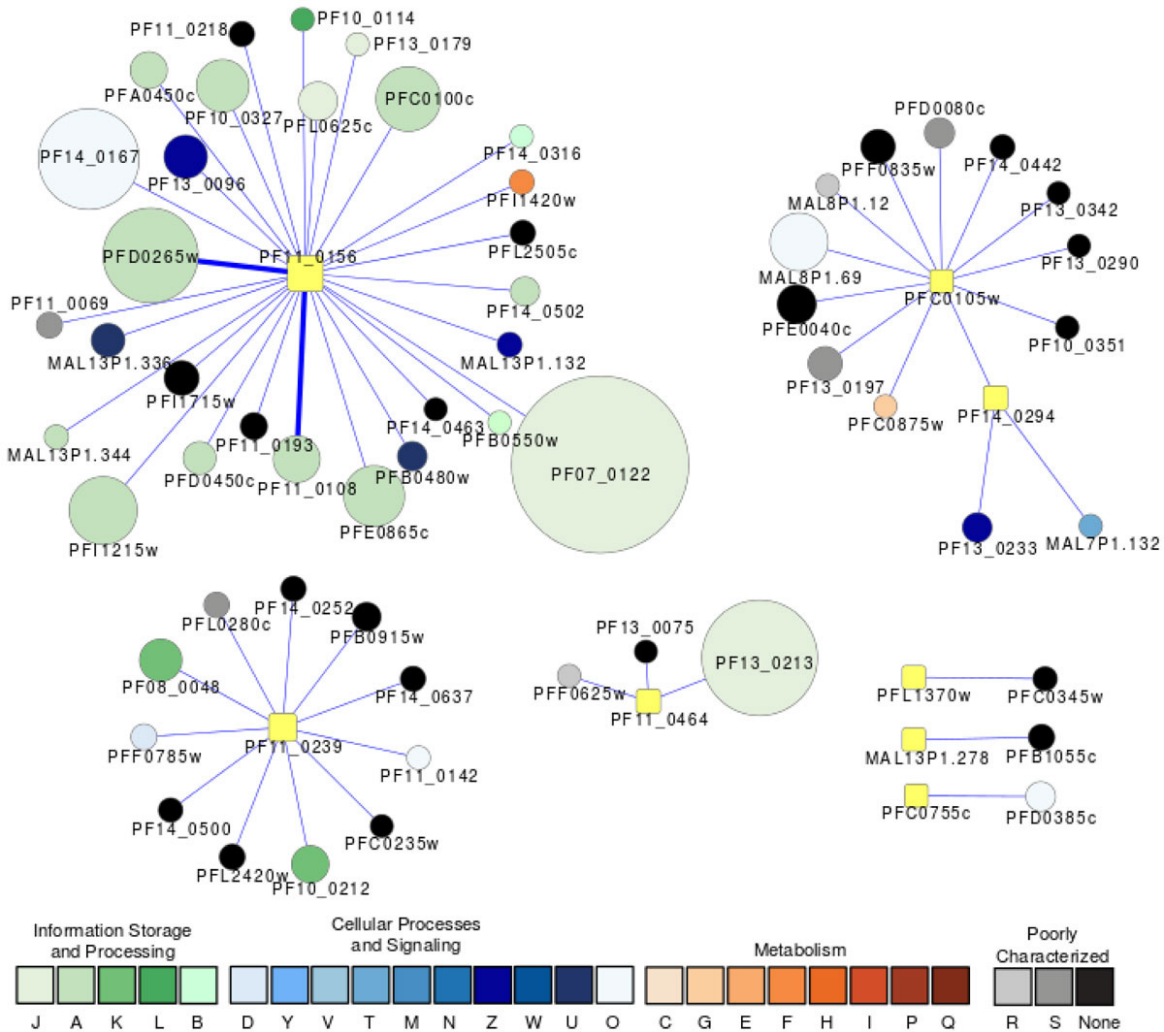
**A graph showing the proteins associated with kinases predicted to be involved in cell cycle regulation**. Square nodes represent the kinases. Node size is proportional to the degree of the connectivity of the node. Nodes are colored according to their functional classification in the eggNOG database [79]. The COG categories are [80] (J) Translation, ribosomal structure and biogenesis, (A) RNA processing and modification, (K) Transcription, (L) Replication, recombination and repair, (B) Chromatin structure and dynamics, (D) Cell cycle control, cell division, chromosome partitioning, (Y) Nuclear structure, (V) Defense mechanisms, (T) Signal transduction mechanisms, (M) Cell wall/membrane/envelope biogenesis, (N) Cell motility, (Z) Cytoskeleton, (W) Extracellular structures, (U) Intracellular trafficking, secretion, and vesicular transport, (O) Posttranslational modification, protein turnover, chaperones, (C) Energy production and conversion, (G) Carbohydrate transport and metabolism, (E) Amino acid transport and metabolism, (F) Nucleotide transport and metabolism, (H) Coenzyme transport and metabolism, (I) Lipid transport and metabolism, (P) Inorganic ion transport and metabolism, (Q) Secondary metabolites biosynthesis, transport and catabolism, (R) General function prediction only, and (S) Function unknown. Confidence scores for the interactions among the nodes (S values from STRING [81]) were divided into three groups - low (0.150-0.399), medium (0.400-0.700) and high (0.701-0.999); the groups are represented by thin, medium and heavy lines, respectively.

Signal transduction plays a key role in managing the complexity of the cell cycle [59,60]. Figure 1 shows eight kinases (in yellow) that were predicted by the subnetwork alignments and the proteins that are directly associated with them. Three protein kinases have been implicated in cell cycle regulation:

(1) PfMAP1 (PF14_0294) is a homolog of mitogen-activated protein kinase (MAPK) [61]. This kinase is believed to be a central member of the MAPKKK cascade and may be related to parasite responses to a variety of exogenous or endogenous stimuli or environmental stresses. PfMAP1 has three PPI partners: (a) a serine/threonine protein kinase (SRPK1) (PFC0105w). PfSRPK plays a role in mRNA splicing machinery [62]. Gene disruption of SRPK in the rodent parasite *P. berghei *suggested that it is essential during male gamete formation [63]. (b) myosin A (PF13_0233) is a component in the linear motor that promotes merozoite motility in invasion. (c) MAL7P1.132, a conserved *Plasmodium *protein of unknown function. This protein was recently annotated as a putative kinase [64].

(2) PfNek-1(PFL1370w) encodes a NIMA-related kinase and it is considered to be a potential antimalarial target. A recent study based on reverse genetics showed that it is required for the asexual cycle in red blood cells and it has sexual specificity (expression in male gametocyte) [65]. PfNek-1 is shown by yeast 2-hybrid assay to pool with a conserved hypothetical protein PFC0345w. Both proteins have abundant expression at the schizont stage.

(3) cdc2-related protein kinase 4 (CRK4) (PFC0755c) [57], was observed as a phospho-protein in the schizont stage of *P. falciparum*-infected red blood cells. Y2H showed that it has a direct interaction with an AAA family ATPase.

The most highly connected kinase predicted to be involved in the cell cycle is the serine/threonine protein kinase PfCLK-3 (PF11_0156) with 28 association partners. Ten proteins were pooled by Y2H experiments [37], including a rhoptry neck protein 3 (RON3), a splicing factor 3A subunit, eukaryotic translation initiation factor 3 subunit 10, a chloroquine resistance marker protein (CRMP), syntaxin involved in vesicle exocytosis, an export protein, and five conserved hypothetical proteins, indicating PfCLK-3's involvement in merozoite invasion, splicing, translation and trafficking. Global kinome analysis suggested that PfCLK3 is likely to be essential for parasite schizogony in RBCs [28].

A calcium-dependent protein kinase 6 (PfCDPK6) (PF11_0239) was predicted to be involved in cell cycle regulation by subnetwork alignment. Previous phenotypic analysis showed that CDPK6 plays a role in sporozoite formation and invasion of hepatocytes [66]. This kinase is associated with 11 other proteins verified by Y2H assays. Two of the association partners are likely involved in cell cycle regulation as well: a putative Ndc80 protein functions in spindle checkpoint signaling for kinetochore organization and movements, and a putative Snf2-related CBP activator (SRCAP) for base excision repair and chromosome remodeling. PfCDPK6 is also associated with PfBet1 in SNARE complex for secretion, a putative protein localized to rhoptry that might be related to merozoite invasion process, a liver-stage antigen, a ubiquitin domain containing protein, and five hypothetical proteins.

The functional roles of other predicted kinases are largely unknown. PF11_0464 is a putative serine/threonine protein kinase. A gene disruption attempt suggested that it is likely essential for the parasite RBC stage [28]. This protein is associated with two proteins required for 60S ribosomal subunit biogenesis (60S ribosomal protein L6-2 and nucleolar GTP-binding protein 1), and a pseudogene of surface-associated interspersed gene 13.1 (SURFIN13.1), which was implicated in the invasion process. MAL13P1.278 (PfArk3) is a putative serine/threonine kinase in the aurora-related kinase (ARK) family. This family of kinases has been implicated in regulation of endocytosis and of the actin skeleton [67]. PfArk3 has a weak association with an erythrocyte membrane protein 1, PfEMP1 (PFB1055) that may be related to mitotic recombination.

#### 3. Proteins implicated in cell division, chromosome organization, and DNA replication

**Figure 2:**
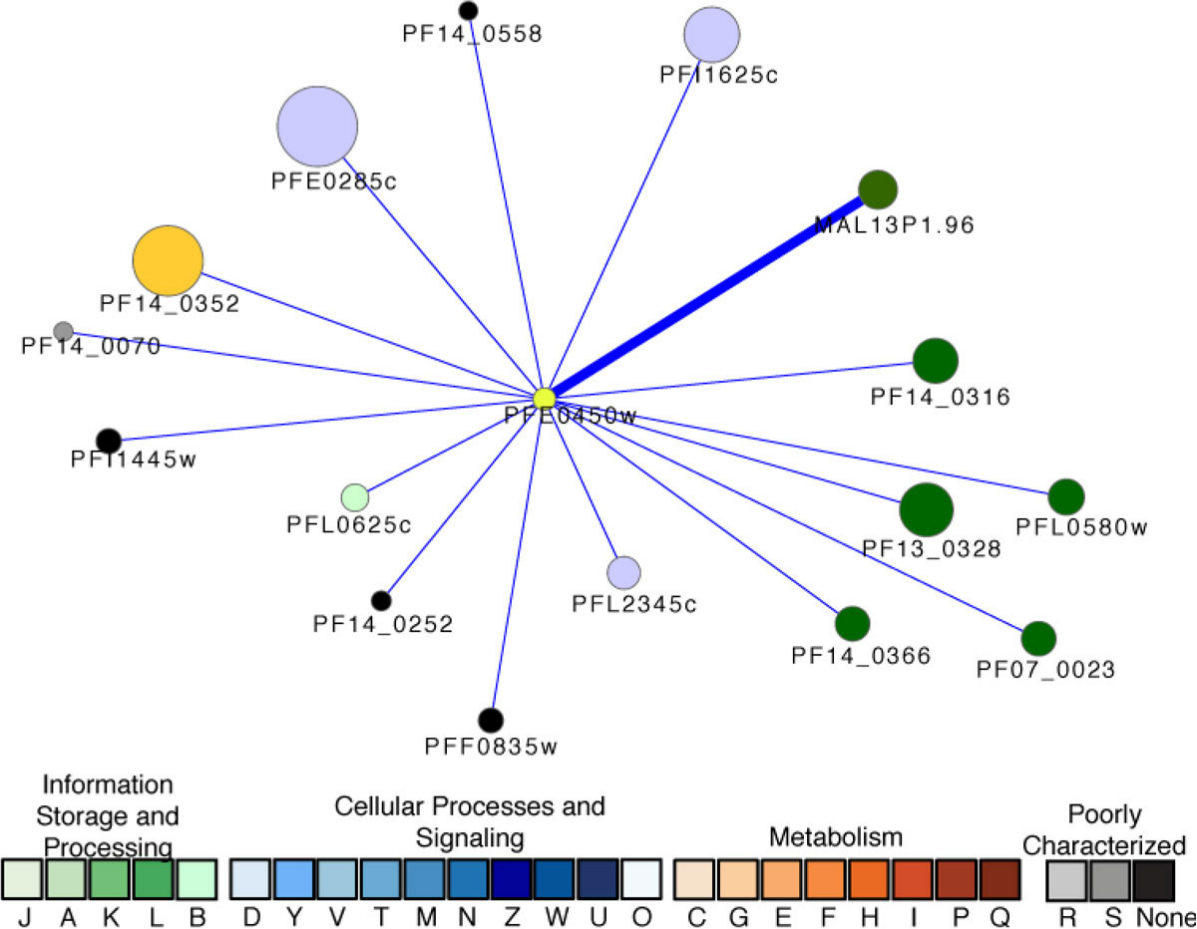
**The proteins associated with a putative chromosome condensation protein PFE0450w**. Node size is proportional to the degree of the connectivity of the node. The visualization is as for Figure 1.

Our analysis has implicated a number of other predicted proteins in the cell division, mitosis, chromosome organization, and DNA replication processes. PFE0450w, a putative chromosome condensation protein that forms part of the ATP-dependent chromatin remodeling complex [68], was predicted to be associated with cell cycle regulation. As shown in Figure 2, are 16 proteins associated with PFE0450w. Eight of these associations have been verified by Y2H, a set that includes two tat-binding proteins pertinent to proteasome activities, a pre-mRNA splicing factor, an eukaryotic translation initiation factor 3 subunit 10, and three conserved *Plasmodium *proteins with unknown function. Perhaps the most important association suggested by our analysis is its link with the high molecular weight rhoptry protein 2 (RhopH2). Rhop2 is localized in the rhoptries of schizonts and plays a role in cytoadherence and merozoite invasion of the red blood cell [69]. Several key components including DNA replication licensing factors and an origin recognition complex subunit were predicted by our subnetwork alignment.

#### 4. DNA repair proteins

**Figure 3:**
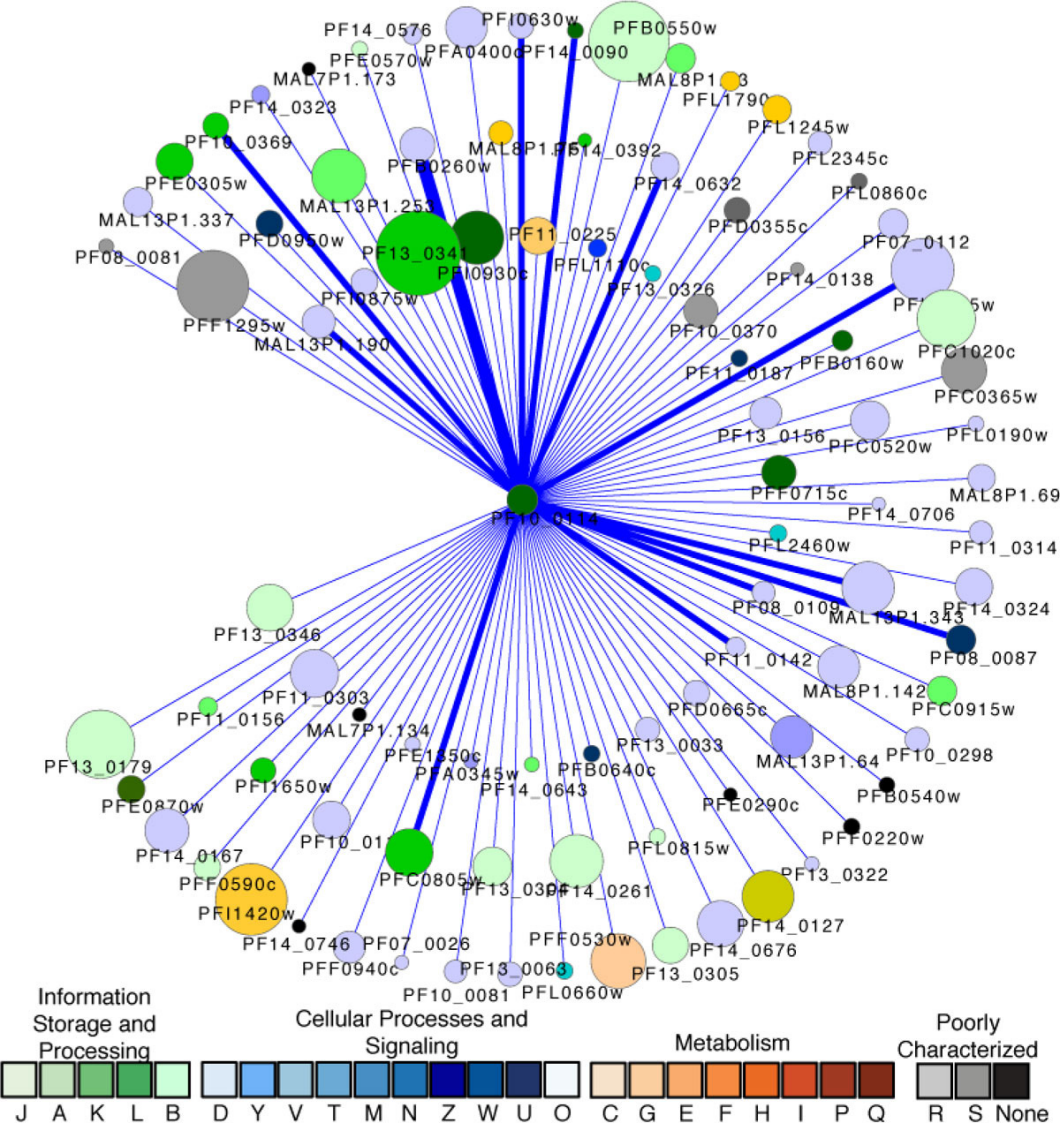
**The proteins associated with a putative DNA repair protein RAD23 (PF10_0114)**. Node size is proportional to the degree of the connectivity of the node. Nodes are colored according to their functional classification in the eggNOG database. The visualization is as for Figure 1.

The cell cycle is also involved in involving DNA repair mechanisms that ensure genome integrity. A putative DNA repair protein RAD23 (PF10_0114) was predicted to have 92 protein-protein association partners (Figure 3), 22 of which have been demonstrated to be direct Y2H physical interactions. This protein is a member of an escort complex for proteasome-mediated degradation of non-native ER proteins. Other suggested interactors with RAD23 include heat shock chaperone proteins, ATP-dependent proteases, serine-threonine kinases, and secreted proteins that have been implicated in stress responses, signaling cascades, and protein sorting and trafficking.

#### 5. Transcriptional regulators

**Figure 4:**
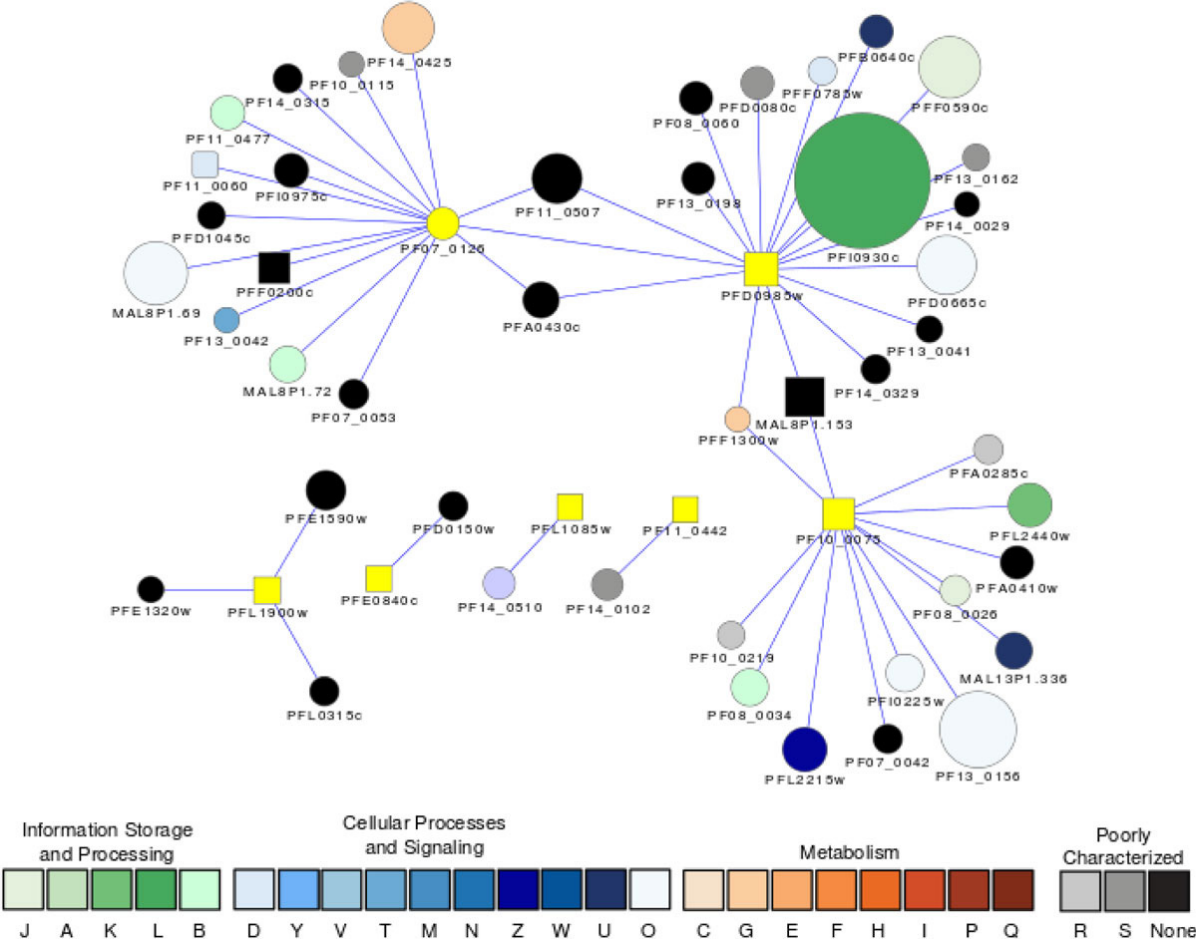
**The proteins associated with ApiAP2 transcriptional regulators**. Square nodes represent the ApiAp2 proteins. Node size is proportional to the degree of the connectivity of the node. Nodes are colored according to their functional classification in the eggNOG database. The visualization is as for Figure 1.

Seven parasite-specific ApiAP2 transcription factors were predicted to have a role in cell cycle regulation, underscoring the importance of transcriptional regulation. ApiAP2 proteins are gaining recognition as attractive drug targets due to their critical roles in the parasite life cycle and their distant evolutionary relationship to the host, implying a diminished possibility of side-effects for humans [70]. The ApiAP2 protein with the highest degree of connectivity in the cell cycle regulatory network is PFD0985w (Figure 4). Its 17 association partners play versatile roles in epigenetic regulation, kinetochore organization, host cell entry and adhesion, secretion, and protein degradation by the ubiquitin-proteasome system [45]. The roles of another ApiAP2 protein (PF07_0126) can be inferred from its associations with 15 proteins that are related to transcriptional regulation, chromatin remodeling, replication, and repair. This protein has interactions with multiple signaling molecules including a calcium-dependent protein kinase and a ligand protein in the 14-3-3 family.

The involvement of PF10_0075 in ApiAP2 in cell cycle regulation is indicated by its Y2H interactions with another ApiAp2 protein (MAL8P1.153), a histone acetyltransferase GCN5 (PF08_0034), which is important for histone modification and chromatin remodeling [71], a DNA excision repair protein rhp16 (PFL2440w), actin (PFL2215w) and a putative kelch protein whose ortholog was implicated in cytoskeletal function in Atlantic horseshoe crab, *Limulus polyphemus *[72] (Figure 4).

#### 6. Surface antigens

A group of surface antigens in the *Plasmodium falciparum *erythrocyte membrane protein (PfEMP1) family (Table 1) were predicted to be associated with the cell cycle. Encoded by the var gene, PfEMP1 is one of the most abundant protein families in *P. falciparum*. Its polymorphic nature leads to antigenic variation, allowing the parasite to successfully evade the human immune systems, thus contributing to pathogenicity and virulence.

## Conclusions

We have previously developed a neighborhood subnetwork alignment approach and here we apply this method to predict the network components involved in cell cycle regulation. The network components identified included cyclins, kinases, transcriptional regulators, and cell surface antigens, among others. Some of these are obvious and have already been confirmed by experimental approaches, such as yeast two-hybrid experiments. This validates our approach as a useful tool for *in silico *prediction of previously unrecognized interactors in cell cycle regulation and suggests that the expanded set of interactors discussed here form a new set of potential targets for drugs or therapies.

## Methods

### Subnetwork querying by neighborhood alignments

The prediction of functional orthologs for the *P. falciparum *proteins has been structured as a subnetwork querying problem. *Network Querying *is a technique that searches a large "target" network of an organism to find subnetwork regions that look similar to a given query network of another organism [73,74]. The "query" network that we are searching against "target" network is the well-studied functional module in a model organism. Network Querying allows us to predict similar modules in the less studied target organism, providing a way to relate biological knowledge of functionality across organisms [75]. Previously, we applied a neighborhood alignment method for subnetwork querying to predict novel transcriptional regulators with versatile roles in the parasite life cycle [43]. We adopted the same method to identify proteins involved in cell cycle regulation.

First a set of proteins related to cell cycle regulation (GO:0007049: cell cycle) in *E. coli *were mapped onto the its own PPI network. For each cell cycle protein a set of "neighbors" was selected, creating a subnetwork, and by inference, a network of subnetworks in the query network. Conversely using the same technique, each *P. falciparum *protein was mapped into its own PPI network, and a subnetwork of neighbors was constructed. To construct neighborhood subnetworks of comparable size for alignment, proteins that are *k *hops from the central were included and *k *was chosen such that the neighbor size was under 500, unless the central protein had more than 500 neighbors.

After obtaining the neighborhood subnetworks for both the *E. coli *cell cycle proteins and the *P. falciparum *proteins, the *E. coli *subnetworks were combinatorically aligned against the *P. falciparum *subnetworks. The central protein of the best-aligned *P. falciparum *subnetwork was labeled a functional ortholog of the proteins involved in cell cycle regulation in *E. coli *.

**Figure 5:**
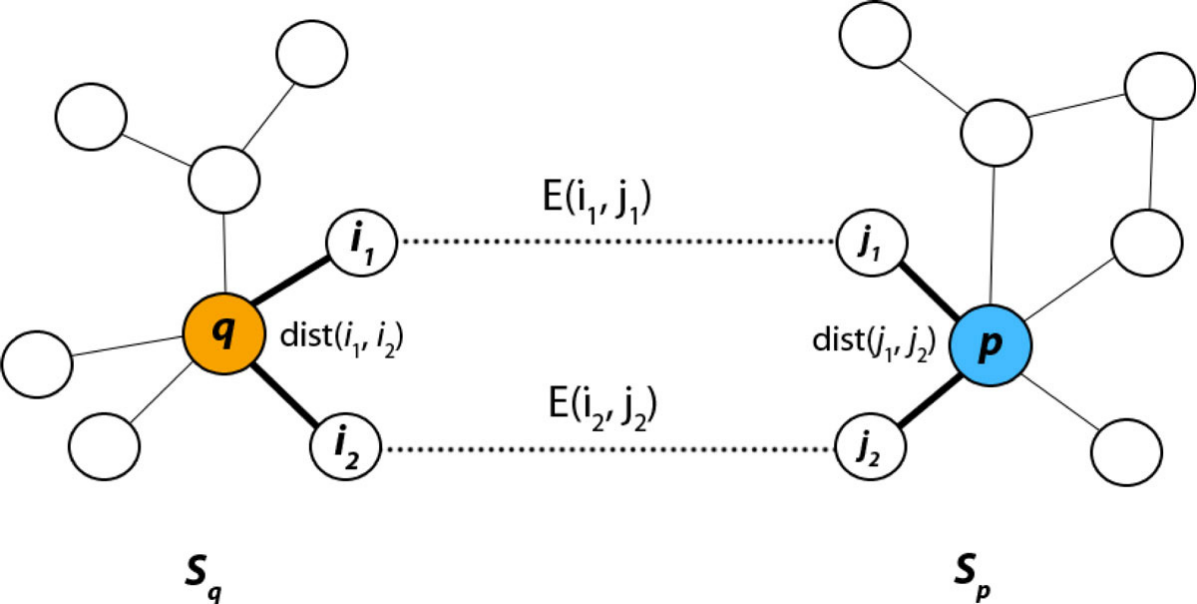
**Subnetwork alignment**. See Methods section for the description of the algorithm.

Analysis to determine how well the *P. falciparum *neighborhood subnetworks aligned with the *E. coli *neighborhood subnetworks was done by assigning a numerical score for each alignment by a shortest-path graph kernel to measure the similarity between two labeled networks [76]. To optimize the graph kernel for this specific use case; only paths between the central protein and other subnetwork proteins are counted. Each shortest path through the central protein characterizes the functional role of the protein in the chained molecular activities along the path. As shown in Figure 5, given two subnetworks *S_p _*with central protein *p *and *S_q _*with central protein *q*, the shortest path similarity function is defined as follows,

$$K\left(S_{q},S_{p}\right)=\frac{1}{\left|S_{q}\right|+\left|S_{p}\right|}\underset{\forall \left(i1,i2\right)\in S_{q}}{\prod }B\left(\left(i1,i2\right)S_{p}\right)$$

Where

$$B\left(\left(i1,i2\right),S_{p}\right)=\underset{\forall \left(j1,j2\right)\in S_{p}}{\text{max}}\frac{2E\left(i1,j1\right)E\left(i2,j2\right)}{dist\left(i1,i2\right)+dist(j1,j2),}$$

$E\left(x,y\right)=exp\left(-\frac{Eval\left(x,y\right)}{\sigma }\right)$ with the normalization parameter σ=10 measures the sequence similarity between proteins × and y based on the E-value of the sequence alignment, and dist(x,y) is the length of the shortest path connecting proteins  x and  y in the PPI subnetwork. The computation was done on a $-{\text{log}}_{10}$ scale. The method outlined here takes each pair of proteins (i1,i2) from one subnetwork and seeks the maximum ratio of sequence similarity with respect to the closeness (shortest path through the central protein) of the networks, in order to identify proteins (j1,j2) in the target subnetwork. From this algorithm, a subnetwork alignment score is obtained by, collecting the shortest paths between two neighborhood subnetworks, getting an alignment score for each pair of proteins, and totaling all of the alignment values. This approach allows for the summarization of the functional coherence, and distance between two central proteins, into a numerical score by way of evaluating the sequence similarity and the role of the central protein between two subnetworks.

**Figure 6:**
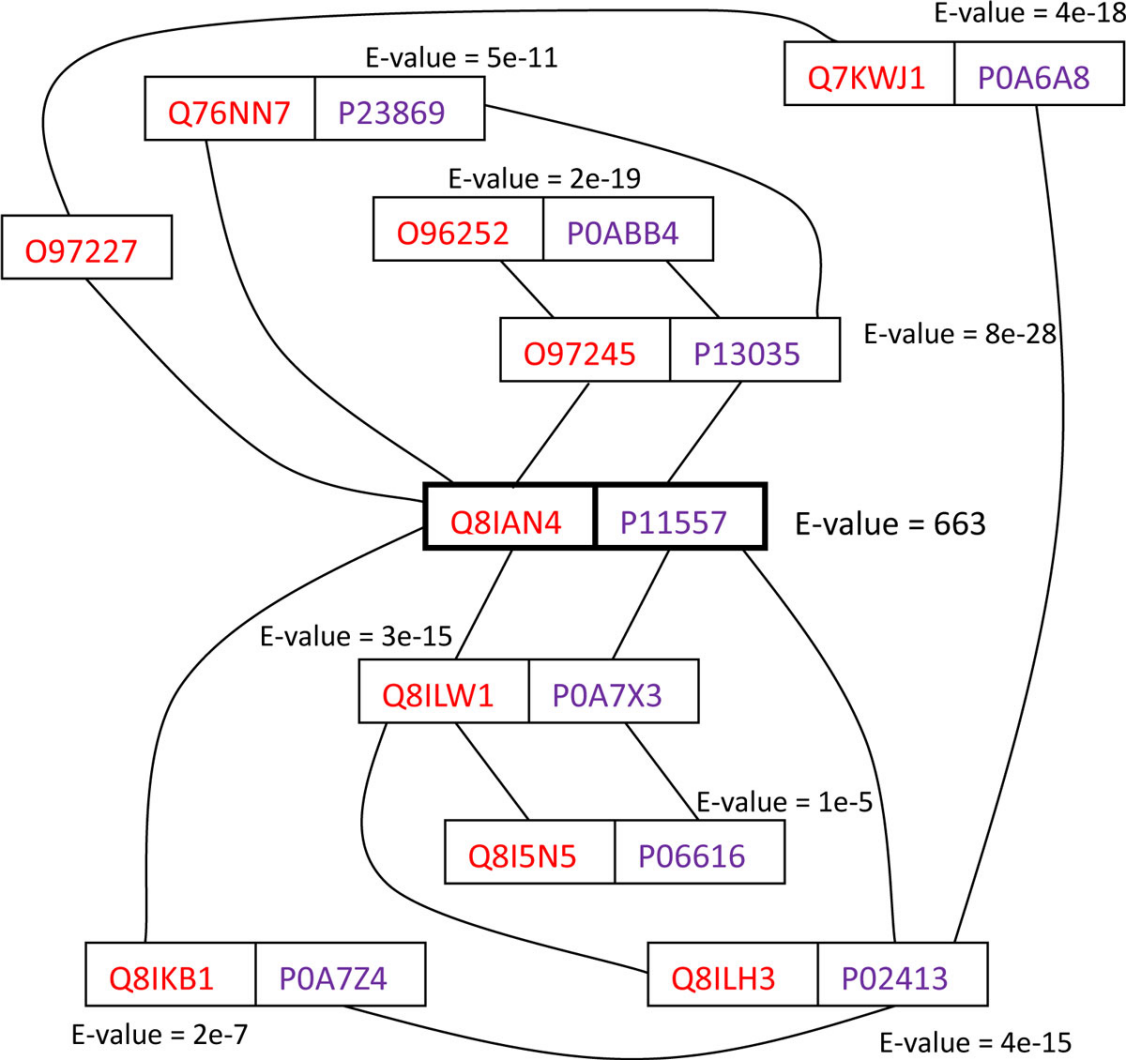
**An example of functional orthologs predicted by subnetwork alignment**. A subnetwork alignment between *E. coli *(proteins labeled in blue) and *P. falciparum *(proteins labeled in red). Because the subnetworks are similar and composed almost entirely of proteins with low BLAST E-values, that is, homologous pairs, it is likely that Q8IAN4 and P11557 are functional homologs, despite their low sequence similarity.

An example of how the subnetwork alignment approach is used to predict functional orthologs is shown in Figure 6 (annotations are shown in Additional File 2). Although the *P. falciparum *protein encoded by locus PF08_0126 (Uniprot ID Q8IAN4, a putative DNA repair protein rad54) and *E. coli *protein DamX (P11557) showed no significant homology, they did share eight pairs of sequence and network orthologs when their PPI networks were aligned. DamX has been shown to directly or indirectly interfere with cell division in *E. coli *[77,78]. Despite their low sequence similarity (BLAST E-value 663), the network alignment evidence suggests that DamX and Q8IAN4 are likely to be functional orthologs.

### Data preparation and network analysis

Protein-protein interaction data for *E. coli *were downloaded from the IntAct database [44]. Protein association data for *P. falciparum *were extracted from the STRING database [45]. STRING assigns association confidence scores (S), ranging from 0.15 to 0.999, based on sequence similarity, pathway analysis [24,46], chromosome synteny, genome organization, phylogenetic reconstruction, and literature text mining. Cytoscape 2.8.3 was used for network visualization [47]. Nodes are colored according to their functional classification in the eggNOG database [48]. NetworkAnalyzer was used to compute topological parameters of the networks [49], with the default settings. Gene Ontology (GO) enrichment analysis was conducted using BiNGO [50]. The hypergeometric test was used with the Benjamini and Hochberg false discovery rate (FDR) correction with a significance level of 0.05.

## List of abbreviations used

ARK: aurora-related kinase; ASP: apical sushi protein; CRMP: chloroquine resistance marker protein; GO: Gene Ontology; MAPK: mitogen-activated protein kinase; PPI: protein-protein interaction; RBC: red blood cell; VBEM: variational Bayesian expectation maximization; Y2H: yeast 2-hybrid.

## Competing interests

The authors declare that they have no competing interests.

## Authors' contributions

YW and RK conceived and designed the study. All authors performed bioinformatics data analysis and drafted the manuscript. All authors read and approved the final manuscript.

## Supplementary Material

Additional file 1 **Functional orthologs involved in cell cycle regulation in *P. falciparum*. **The query genome is *P. falciparum*, and the target genome is *E. coli*. GO: Gene Ontology. BP: Biological Process. MF: Molecular Function. CC: Cellular Component. Click here for file

Additional File 2 **An example of functional orthologs predicted by subnetwork alignment**. The predicted pair is shaded. Click here for file

## Declarations

The publication costs for this article were funded by the corresponding author (YW).

This article has been published as part of BMC Bioinformatics Volume 14 Supplement 12, 2013: Selected articles from the IEEE International Conference on Bioinformatics and Biomedicine 2012: Bioinformatics. The full contents of the supplement are available online at http://www.biomedcentral.com/bmcbioinformatics/supplements/14/S12.
