# Supplementary material for: A novel subnetwork alignment approach predicts new components of the cell cycle regulatory apparatus in Plasmodium falciparum
Source: BMC Bioinformatics. 2013 Sep 24;14(Suppl 12):S2. doi: 10.1186/1471-2105-14-S12-S2 (PMC3848769; doi:10.1186/1471-2105-14-S12-S2)
Supplement: Additional File 2 — An example of functional orthologs predicted by subnetwork alignment. The predicted pair is shaded. [file 1471-2105-14-S12-S2-S2.docx]

**Additional File 2: An example of functional orthologs predicted by subnetwork alignment.** The predicted pair is shaded.

| *P. falciparum* proteins | | | *E. coli* proteins | | Blast E-value |
| --- | --- | --- | --- | --- | --- |
| Uniprot ID | PlasmoDB ID | Annotation | Uniprot ID | Annotation |  |
| Q8IAN4 | PF08_0126 | Putative DNA repair protein rad54 | P11557 | Protein DamX | 663 |
| O97245 | PFC0275w | FAD-dependent glycerol-3-phosphate dehydrogenase | P13035 | Aerobic glycerol-3-phosphate dehydrogenase | 8e-28 |
| Q8ILW1 | PF14_0132 | Putative 40S ribosomal protein S9A | P0A7X3 | 30S ribosomal protein S9 | 3e-15 |
| Q76NN7 | PFC0975c | peptidyl-prolyl cis-trans isomerase (CYP19A) | P23869 | Peptidyl-prolyl cis-trans isomerase B | 5e-11 |
| Q8IKB1 | PF14_0695 | Putative DNA-directed RNA polymerase, alpha subunit | P0A7Z4 | DNA-directed RNA polymerase subunit alpha | 2e-7 |
| O96252 | PFB0795w | ATP synthase F1, alpha subunit | P0ABB4 | ATP synthase subunit beta | 2e-19 |
| Q8I5N5 | PFL0835w | Putative GTP binding protein | P06616 | GTPase Era | 1e-5 |
| Q8ILH3 | PF14_0270 | Putative apicoplast ribosomal protein L15 precursor | P02413 | 50S ribosomal protein L15 | 4e-15 |
| Q7KWJ1 | PFB0385w | acyl carrier protein (ACP) | P0A6A8 | Acyl carrier protein | 4e-18 |
